# Effective interventions to facilitate the uptake of breast, cervical and colorectal cancer screening: an implementation guideline

**DOI:** 10.1186/1748-5908-6-112

**Published:** 2011-09-29

**Authors:** Melissa C Brouwers, Carol De Vito, Lavannya Bahirathan, Angela Carol, June C Carroll, Michelle Cotterchio, Maureen Dobbins, Barbara Lent, Cheryl Levitt, Nancy Lewis, S Elizabeth McGregor, Lawrence Paszat, Carol Rand, Nadine Wathen

**Affiliations:** 1 Program in Evidence-based Care, Cancer Care Ontario, Hamilton, Ont., Canada; 2 Departments of Oncology and Clinical Epidemiology and Biostatistics, McMaster University, Hamilton, Ont., Canada; 3 Hamilton Urban Core Community Centre, Hamilton, Ont., Canada; 4 Department of Family and Community Medicine, Mount Sinai Hospital, University of Toronto, Toronto, Ont., Canada; 5 Population Studies and Surveillance, Cancer Care Ontario, Toronto, Ont., Canada; 6 School of Nursing, McMaster University, Hamilton, Ont., Canada; 7 Department of Family Medicine, The University of Western Ontario, London, Ont., Canada; 8 Department of Family Medicine, McMaster University, Hamilton, Ont., Canada; 9 Primary Care, Cancer Care Ontario, Toronto, Ont., Canada; 10 Prevention and Screening, Cancer Care Ontario, Toronto, Ont., Canada; 11 Population Health Research, Alberta Health Services - Cancer Epidemiology, Prevention and Screening, Calgary, Alb., Canada; 12 Department of Health Policy Management and Evaluation, University of Toronto, Toronto, Ont., Canada; 13 Department of Radiation Oncology, University of Toronto, Toronto, Ont., Canada; 14 Regional Cancer Prevention and Early Detection Network Hamilton, Niagara, Haldimand, Brant., Canada; 15 Systemic, Supportive and Regional Cancer Programs, Juravinski Cancer Centre, Hamilton, Ont., Canada; 16 Faculty of Information and Media Studies, The University of Western Ontario, London, Ont., Canada

## Abstract

**Background:**

Appropriate screening may reduce the mortality and morbidity of colorectal, breast, and cervical cancers. Several high-quality systematic reviews and practice guidelines exist to inform the most effective screening options. However, effective implementation strategies are warranted if the full benefits of screening are to be realized. We developed an implementation guideline to answer the question: What interventions have been shown to increase the uptake of cancer screening by individuals, specifically for breast, cervical, and colorectal cancers?

**Methods:**

A guideline panel was established as part of Cancer Care Ontario's Program in Evidence-based Care, and a systematic review of the published literature was conducted. It yielded three foundational systematic reviews and an existing guidance document. We conducted updates of these reviews and searched the literature published between 2004 and 2010. A draft guideline was written that went through two rounds of review. Revisions were made resulting in a final set of guideline recommendations.

**Results:**

Sixty-six new studies reflecting 74 comparisons met eligibility criteria. They were generally of poor to moderate quality. Using these and the foundational documents, the panel developed a draft guideline. The draft report was well received in the two rounds of review with mean quality scores above four (on a five-point scale) for each of the items. For most of the interventions considered, there was insufficient evidence to support or refute their effectiveness. However, client reminders, reduction of structural barriers, and provision of provider assessment and feedback were recommended interventions to increase screening for at least two of three cancer sites studied. The final guidelines also provide advice on how the recommendations can be used and future areas for research.

**Conclusion:**

Using established guideline development methodologies and the AGREE II as our methodological frameworks, we developed an implementation guideline to advise on interventions to increase the rate of breast, cervical and colorectal cancer screening. While advancements have been made in these areas of implementation science, more investigations are warranted.

## Introduction

Cancer screening has the capacity to reduce morbidity and mortality from disease [1]. Several international, national, and regional guidelines exist that provide recommendations on which screening manoeuvres are most effective, efficient, and safe, and for which patients or members of the public [2-4]. However, for screening activities to yield benefits, they must be applied. Thus, identification of effective interventions designed to increase screening rates are needed. The Cancer Screening Uptake Expert Panel in partnership with the practice guidelines program of the Ontario cancer system, Cancer Care Ontario's Program in Evidence-based Care, came together to develop an implementation guideline to identify and recommend appropriate interventions to increase the uptake of screening for breast, cervical, and colorectal cancers (CRCs). Our guidance is intended for healthcare providers, system leaders, and organizations responsible for implementing cancer screening programs and members of the public.

The specific guideline question we asked was: What interventions have been shown to increase the uptake of cancer screening by individuals, specifically for breast, cervical, and CRCs?

Interventions of interest include:

1. Population-based interventions aimed to increase the demand for cancer screening, including client reminders, client incentives, mass media, small media, group education, and one-on-one education.

2. Population-based interventions aimed to reduce barriers to obtaining screening, including reduction in structural barriers and reduction in out-of-pocket costs.

3. Provider-directed interventions targeted at clinicians to implement in the primary care setting, including provider assessment and feedback interventions and provider incentives.

Our outcome of interest was completed screening rates.

## Methods

Cancer Care Ontario's Program in Evidence-based Care and Provincial Screening Program established the Cancer Screening Uptake Panel to complete the guideline project (see Additional file 1: Appendix). The multidisciplinary panel was comprised of primary care providers, researchers, managers of screening programs, experts in implementation science, systematic review, and practice guideline development, and methodologists. Patients with cancers or citizens were not recruited to be on the panel. All panel members disclosed conflicts of interest. No conflicts were identified.

The guideline development cycle [5,6] and the AGREE II framework [7-9] were used as the methodological foundations for this project. The Cancer Screening Uptake Expert Panel conducted an initial scoping review and systematic review that yielded several candidate-synthesized documents that could serve as the evidentiary base for these guideline recommendations. Three systematic reviews [10-12], published in a 2008 special issue of the *American Journal of Preventive Medicine*, were chosen because of their direct relevance to the project objectives, their currency, and their quality. These were accompanied by recommendations from the United States (US) Task Force on Community Preventive Services [13].

A two-stage update process of the systematic reviews was undertaken by the expert panel to identify new eligible studies published between 2004 and May 2010. Quality appraisal of the new studies was untaken. Complete methodological details and results of the systematic reviews can be found elsewhere [14,15].

Together with the evidence from the original reviews [10-12], the expert panel considered the studies over several meetings and came to a consensus on a set of guideline recommendations, suggestions for how the recommendations could be used, and ideas for future research priorities. The panel considered issues of data quantity, data quality, and the Ontario context when interpreting and judging the evidence. A decision rubric was developed by the panel, informed by the original reports, in order to have a common language by which the recommendations could be classified. In cases where there was an absence of evidence, the conclusion of the panel was that there was no evidence to refute or support the particular intervention; in cases where the evidence was not compelling, the conclusion was that there was insufficient evidence; in cases where the evidence was compelling, as deemed by the panel, the conclusion was to recommend the intervention. This strategy aligns conceptually with the original reports [10-12]. Consensus was reached by all members of the panel in both the interpretation and classification of recommendations, and no formal consensus method (*e.g*., Delphi technique) were used.

Subsequently, a two-step review strategy was undertaken. First, a draft document was circulated to Cancer Care Ontario's Report Approval Panel (RAP). It is comprised of clinicians, screening experts, and methodologists. Their role was review the draft document with special attention to methodological quality, provide feedback, and ultimately approve the document for circulation to the external reviewer pool.

The revised and approved draft document was then circulated to eight Canadian and American external reviewers with expertise in the clinical and methodological aspects of cancer screening and guideline development. They were asked to complete questionnaire using a five-point scale targeting the quality of guideline (higher scores indicating better quality) and their intention to use a guideline of this quality (higher scores indicating greater intention). Specifically, these stakeholders rated the guideline development methods, guideline presentation, guideline recommendations, completeness of recommendations, whether there was sufficient information to inform decisions, overall guideline quality, the likelihood they would make use of this guideline in professional decisions, and in their practice. Note that the psychometric properties of this questionnaire have not been fully tested. The expert panel took this feedback and made revisions before the final document was released to Cancer Care Ontario [14].

The project was funded by Cancer Care Ontario though the Ontario Ministry of Health and Long-Term Care. The guideline was editorially independent from the funding source.

## Results

### Evidentiary base

An additional 66 randomized controlled trials (RCTs) and cluster randomized trials reflecting 74 comparisons were found that met inclusion criteria. Overall, the quality of the trials ranged considerably, but was generally weak. Full details on the results of the updates can be found elsewhere [14]. Thus, three foundational systematic reviews [10-12], additional trial data [16-89] and the original recommendations of the US Task Force [13] served as the evidentiary foundation to inform the guideline recommendations reported here. A draft practice guideline document was crafted. The first section consisted of the guideline questions, statement of the intended users, overview of the key evidence, draft recommendations, qualifying statements, advice on how to use the recommendations, and research priorities.

### Draft guideline review

The RAP review of the draft guideline document yielded favourable feedback for the expert panel. Key modifications included strengthening the section on how the guidance should be used and being more explicit about some the challenges inherent the implementation science literature. With respect the external review process, feedback was received from five reviewers. Mean ratings were favourable across all indices: quality of methods (4.8), guideline presentation (4.6), quality of recommendations (4.2), completeness of reporting (4.8), sufficient information to inform decisions (4.2), overall quality (4.6), intention to use (4.8), and willingness to recommend for use in practice (5.0) (range 1 to 5; 5 most favourable score). Reviewers provided suggestions for how the recommendations could be implemented. Final revisions to the guideline were made.

### Recommendations

Table 1 summarizes the recommendations of the Cancer Screening Uptake Expert Panel regarding population-based interventions to increase the demand for cancer screening, population-based interventions to reduce barriers to obtaining screening, and provider-directed interventions targeted at clinicians to implement in the primary care setting.

**Table 1:** Summary of recommendations of cancer care Ontario's Cancer Screening Uptake Expert Panel for breast, cervical and colorectal cancer screening

Population-based interventions to increase demand for screening				References
*Intervention*	*Breast*	*Cervical*	*Colorectal*	
Client reminders	Recommended			[16-33]
Client incentives alone	There is insufficient evidence to recommend for or against this intervention.			
Mass media alone	There is insufficient evidence to recommend for or against this intervention.			[34-56]
Small media	Recommended			

Group education	There is insufficient evidence to recommend for or against this intervention.			[57-74]
One-on-one education	Recommended	Recommended	Consider	

**Population-based interventions to reduce barriers to obtaining screening**				**References**
*Intervention*	*Breast*	*Cervical*	*Colorectal*	
Reducing structural barriers	Recommended	Recommended	There is insufficient evidence to recommend for or against this intervention.	[75-80]
Reducing out-of-pocket costs	There is insufficient evidence to recommend for or against this intervention for the Ontario context.			

**Interventions directed at healthcare providers to increase screening**				**References**
*Interventions*	*Breast*	*Cervical*	*Colorectal*	[81-89]
Provider assessment and feedback	Recommended			
Provider incentives	There is insufficient evidence to recommend for or against this intervention.			

Note: Recommendation table template designed by the United States (US) Task Force on Community Preventive Services [13].

### Specific recommendations

1. Client reminders and small media are effective population-based interventions to increase the uptake of breast, cervical, and CRC screening.

2. One-on-one education is an effective population-based intervention to increase the uptake of breast and cervical cancer screening. Evidence is emerging suggesting one-on-one education might facilitate the uptake of CRC screening, and should be considered as an option in the context of CRC screening.

3. Reducing structural barriers is an effective intervention to increase community access and reduce barriers to breast and cervical cancer screening. There is insufficient evidence to support or refute its role in CRC screening.

4. Provider assessment and feedback is an effective provider-focused intervention to increase the uptake of breast, cervical, and CRC screening.

5. At this time, there is insufficient evidence to support or refute the role of client incentives, mass media, group education, reducing out-of-pocket costs, and provider incentives as strategies to increase the uptake of breast, cervical, or CRC screening.

6. There are no interventions studied in this review that led the Cancer Screening Uptake Expert Panel to recommend unequivocally against their use because of proven ineffectiveness.

With few exceptions, the recommendations of the Cancer Screening Uptake Expert Panel align with the original recommendations of the US Task Force. The exceptions include:

1. The expert panel chose not to categorize the strength of the recommendations or evidence foundation due to the inability to form reliable operational definitions that could be consistently applied across the areas of inquiry.

2. The expert panel believes the new evidence emerging in the update is sufficient to reclassify one-on-one education for CRC from the original 'not recommend' to 'consider' as an option. The 'consider' category emerged *post hoc *after the initial decision rubric was developed.

3. The expert panel did not view the evidence regarding reducing out-of-pocket costs for patients as relevant to the publicly-funded Ontario context and could not recommend for or against that intervention. This may be pertinent to similar contexts in which screening is offered. Covering patient expenditures associated with screening, for example, parking, or colonoscopy preparation material costs are additional strategies worthy of study that may remove barriers that prevent a patient obtaining a screening procedure. Privately-funded systems may interpret these data differently for their context and arrive at different recommendations.

### Qualifying statements

#### Recommendation caveats

1. There is little evidence directly testing the effectiveness of interventions for different populations; nonetheless, subgroup analysis suggests group education may be a useful intervention for special populations such as specific ethnic groups or other groups for whom access to healthcare might be challenging.

2. There is little evidence directly testing the effectiveness of interventions for different provider groups; nonetheless, evidence suggests that provider assessment and feedback may be more effective for trainees than for established practitioners.

3. Types of provider incentives explored in the original systematic review and the updated studies may or may not be generalizable across healthcare contexts. For example, currently in Ontario, there are some financial incentive strategies (for example, fee codes and bonus payments) for screening that should be explored and evaluated more thoroughly.

4. Across the studies, the labelling, categorization, and operationalization of several of the interventions evaluated were inconsistent and overlapping. This precludes recommendations for specific options within the suite of activities the intervention represents. Nonetheless, it is important to the note that across categories where the greatest overlap exists (*i.e*., client reminders, small media, and one-on-one education) the evidence is generally consistent and in favour of the interventions.

5. The methods by which information was tailored varied across studies. As such, no specific advice can be offered in favour of one tailoring strategy over another.

6. The literature is incomplete in differentiating between newly screened and repeat-screened individuals. This precludes making recommendations for each of these population groups.

7. There are several screening options within each cancer site, particularly in the case of CRC screening (fecal occult blood test (FOBT), flexible sigmoidoscopy (FS), and colonoscopy). Studies varied in terms of the types of screening covered, and in no case was an analysis of a specific modality complete. This precludes making specific recommendations for each screening modality within that disease site.

#### Methodological caveats

1. In contrast to the original systematic reviews that included a range of study designs, the update of the literature focused on RCTs and cluster RCTs only.

2. The quality of RCTs and cluster RCTs in the update was poor, primarily due to the incomplete reporting of quality characteristics information in the studies.

3. Measures of the key outcome, percentage point (PP) change, were calculated in the original systematic reviews and the update using various strategies based on the availability of the data. While larger PP changes are more indicative of greater effectiveness, the absolute magnitude of effect cannot be calculated, and comparisons across studies using different data may be misleading.

#### Resources caveat

An update and review of the cost-effectiveness data analysis fell outside the scope of our guideline due to limited resources to conduct more systematic and high quality cost-effectiveness analyses and the interpretation and, without that, the belief of the panel that the data could be reliably generalized to other contexts. Nonetheless, appropriate planning and resource estimates should be considered before the implementation of an intervention.

#### How to apply the recommendations

The recommendations provide information regarding what suites of interventions are more or less effective at increasing the uptake of cancer screening. The recommendations do not provide specific advice regarding which activity or elements within that intervention group should be implemented or for which specific populations or providers one might see the greatest effect. To make these decisions, users are encouraged to do the following:

1. Choose a few candidate studies with populations, providers, and contexts that most closely align with your own populations, providers, and context.

a. This can be accomplished by reviewing study details presented in the text and tables in the systematic reviews [14,15].

b. Recognize that there is significant overlap across some of the intervention categories that show the greatest promise (*e.g*., client reminders, one-on-one education, and small media), and consider this when developing your own suite of interventions.

2. Consider and deliberate:

a. Which activities and operational details have the greatest face validity for your context?

b. Would these activities be acceptable to the populations you are targeting?

c. Do you have the resources (*e.g*., human, financial) to offer these interventions?

d. Do you have the capacity to measure their impact?

3. Contribute to the knowledge base.

4. Where possible, build into your activities a formal, high-quality evaluation strategy and communicate your findings to a wider audience, including the scientific community. These data can be used to improve the knowledge base and enable health services researchers to refine what is known and provide more precise recommendations in the future.

#### Potential research areas

The evidence review identified several potential research areas that could advance the knowledge in this area. Some of these include:

1. Research targeting interventions that provide insufficient information to conclude for or against effectiveness. This includes client incentives, mass media, group education, reducing out-of-pocket costs (relevant specific contexts), and provider incentives as strategies to increase the uptake of breast, cervical, or CRC screening.

2. Research to disentangle the multiple operational elements that define the various interventions to test those that are more and less effective, and further, to explain whether the cumulative impact of these interventions can facilitate achieving the desired behavioural outcomes

3. Research specifically designed to study the effects of interventions across different populations. Repeat-screened versus never-been-screened populations, general populations versus specific ethnic groups, and other groups for whom access to healthcare might be more challenging are of particular interest.

4. Research to determine more accurately the efficacy of tailored versus non-tailored approaches, including the cost-effectiveness of more complex tailored approaches.

5. Research to analyze and evaluate the cost-effectiveness of specific interventions using strategies that will yield data relevant to the specific contexts where screening is offered.

6. Research to investigate the impact of more recent electronic and other mass media interventions when targeting either general or specific populations.

7. Research to compare the impact of interventions related to the type of healthcare practitioner delivering that intervention (*e.g*., family physician, nurse practitioner, pharmacist).

## Conclusion

While the guideline was developed within the Ontario (Canada) context, we believe the evidence updates and recommendations provide a valuable source of information to clinicians, policy makers, and researchers internationally who have an interest and mandate to advance quality of cancer control and, in particular, cancer screening. However, we acknowledge that recommendations may vary from jurisdiction to jurisdiction due to the unique system, funding, and contextual structures that may influence the interpretation of evidence and its application [14]. Guideline developers are encouraged to consider formal guideline adaptation strategies in using the systematic review and guideline as a foundation for their own goals (see http://www.cancerview.ca/portal/server.pt/community/tools_and_resources/519/guideline_adaptation/5627) [90].

Our methodology did not include representatives from patient groups and the public. We acknowledge this as limitation. While in the Ontario system the approach is to focus on alternative avenues for engagement of these stakeholders (*e.g*., by regional health networks, implementation committees) these perspectives at the guideline development level would be an asset [91].

Finally, in the field of implementation science, while progress on the research front has been made, further investigation is required and, more importantly, efforts to better direct how research findings can be put into practice are warranted. The panel puts forward suggestions for how best to use the guideline recommendations and areas we saw as future research priorities. Continued advancement in this field are required if the full benefits of cancer screening and their impact on public and patient health will be realized.

## Competing interests

The authors declare that they have no competing interests.

## Authors' contributions

MB and CD developed the original study concept and protocol. MB, CD, and LB were responsible for acquisition and analysis of the data; development of the initial draft manuscript, and manuscript revisions. All authors were responsible for the interpretation of the data; review of the draft versions of the manuscript; provision of feedback for important intellectual revisions; and review and final approval of the version to be published.

## Supplementary Material

Additional file 1 **Appendix 1. Expert panel members**. Appendix 1. Members of Cancer Screening Uptake Expert Panel. Click here for file
