# Supplementary material for: Effective interventions to facilitate the uptake of breast, cervical and colorectal cancer screening: an implementation guideline
Source: Implement Sci. 2011 Sep 29;6:112. doi: 10.1186/1748-5908-6-112 (PMC3222606; doi:10.1186/1748-5908-6-112)
Supplement: Additional file 1 — Appendix 1. Expert panel members. Appendix 1. Members of Cancer Screening Uptake Expert Panel. [file 1748-5908-6-112-S1.PDF]

APPENDIX 1. Members of Cancer Screening Uptake Expert Panel.

Dr. Melissa Brouwers, Chair<br>Provincial Director, Program in Evidence-based Care (PEBC), Cancer Care Ontario<br>Associate Professor, Department of Oncology and Associate Member, Department of Clinical Epidemiology & Biostatics<br>McMaster University, Hamilton ON	Dr. Cheryl Levitt<br>Provincial Clinical Lead, Primary Care<br>Cancer Care Ontario, Toronto ON
Dr. Angela Carol<br>Family Physician<br>Hamilton Urban Core Community Centre<br>Hamilton ON	Dr. Nancy Lewis<br>Senior Policy and Planning Officer<br>Cancer Care Ontario, Toronto ON
Dr. June Carroll<br>Associate Professor<br>Department of Family and Community Medicine<br>Mount Sinai Hospital<br>University of Toronto, Toronto ON	Dr. S. Elizabeth McGregor<br>Research Scientist<br>Population Health Research,<br>Alberta Health Services - Cancer Epidemiology, Prevention & Screening<br>Calgary AB
Dr. Michelle Cotterchio<br>Scientist and Associate Professor<br>Population Studies and Surveillance<br>Cancer Care Ontario<br>and Dalla Lana School of Public Health,<br>University of Toronto, Toronto ON	Dr. Lawrence Paszat<br>Senior Scientist, Institute for Clinical Evaluative Sciences<br>Associate Professor, Dept of Health Policy Management and Evaluation and Dept of Radiation Oncology<br>University of Toronto, Toronto ON
Ms. Carol De Vito<br>Research Coordinator<br>PEBC, Cancer Care Ontario<br>Hamilton, ON	Ms. Carol Rand<br>Chair, Regional Cancer Prevention and Early Detection Network Hamilton, Niagara, Haldimand, Brant<br>Director of Systemic, Supportive and Regional Cancer Programs<br>Juravinski Cancer Centre, Hamilton ON
Dr. Maureen Dobbins<br>Associate Professor and<br>Career Scientist, Ontario Ministry of Health & Long-Term Care<br>School of Nursing, McMaster University<br>Hamilton ON	Dr. Nadine Wathen<br>Associate Professor<br>Faculty of Information & Media Studies<br>University of Western Ontario, London ON
Dr. Barbara Lent<br>Professor<br>Department of Family Medicine<br>University of Western Ontario, London, ON	Ms. Lavannya Bahirathan<br>Research Assistant<br>PEBC, Cancer Care Ontario<br>Hamilton, Ontario
